# TECHNOLOGY DESIGN AND IMPLEMENTATION TO PROMOTE EQUITY, INCLUSION, AND DIVERSITY

**DOI:** 10.1093/geroni/igac059.999

**Published:** 2022-12-20

**Authors:** Walter Boot

## Abstract

The design of technology and technology-based solutions for the challenges many older adults face must consider principles of equity, inclusion, and diversity, or there is a risk of exacerbating digital divides in our society. This session focuses on the design, evaluation, and implementation of technologies to support older adults considering the diversity of the older adult population with respect to factors such as race and ethnicity, income level, health conditions, and cognitive status. M. Harris will discuss the facilitators and barriers identified by Black older adults related to the use of wearable devices for health monitoring. J. Chung will present on the development of a smart speaker application to support wellness among low-income senior housing residents. C. Berridge and E. Sanders will discuss designing technology solutions related to diversity with respect to cognitive status, with Berridge discussing preferences of older adults with mild Alzheimer’s disease for a technology-delivered planning tool, and Sanders presenting technology-based solutions to support the prospective memory of older adults with diverse cognitive impairments. Finally, Schiaffino will present a study examining the acceptability of community-based telehealth programs among vulnerable older adults as a function off health, language, and ethnicity. General themes of inclusive design to ensure that all older adults can benefit from existing and emerging technology-based solutions will be highlighted.

